# Supplementary material for: Microglia are an essential component of the neuroprotective scar that forms after spinal cord injury
Source: Nat Commun. 2019 Jan 31;10:518. doi: 10.1038/s41467-019-08446-0 (PMC6355913; doi:10.1038/s41467-019-08446-0)
Supplement: Supplementary file 1 — Supplementary Information [file 41467_2019_8446_MOESM1_ESM.pdf]

Supplementary Information

Microglia are an essential component of the neuroprotective scar that forms after spinal cord injury.

Bellver et al.

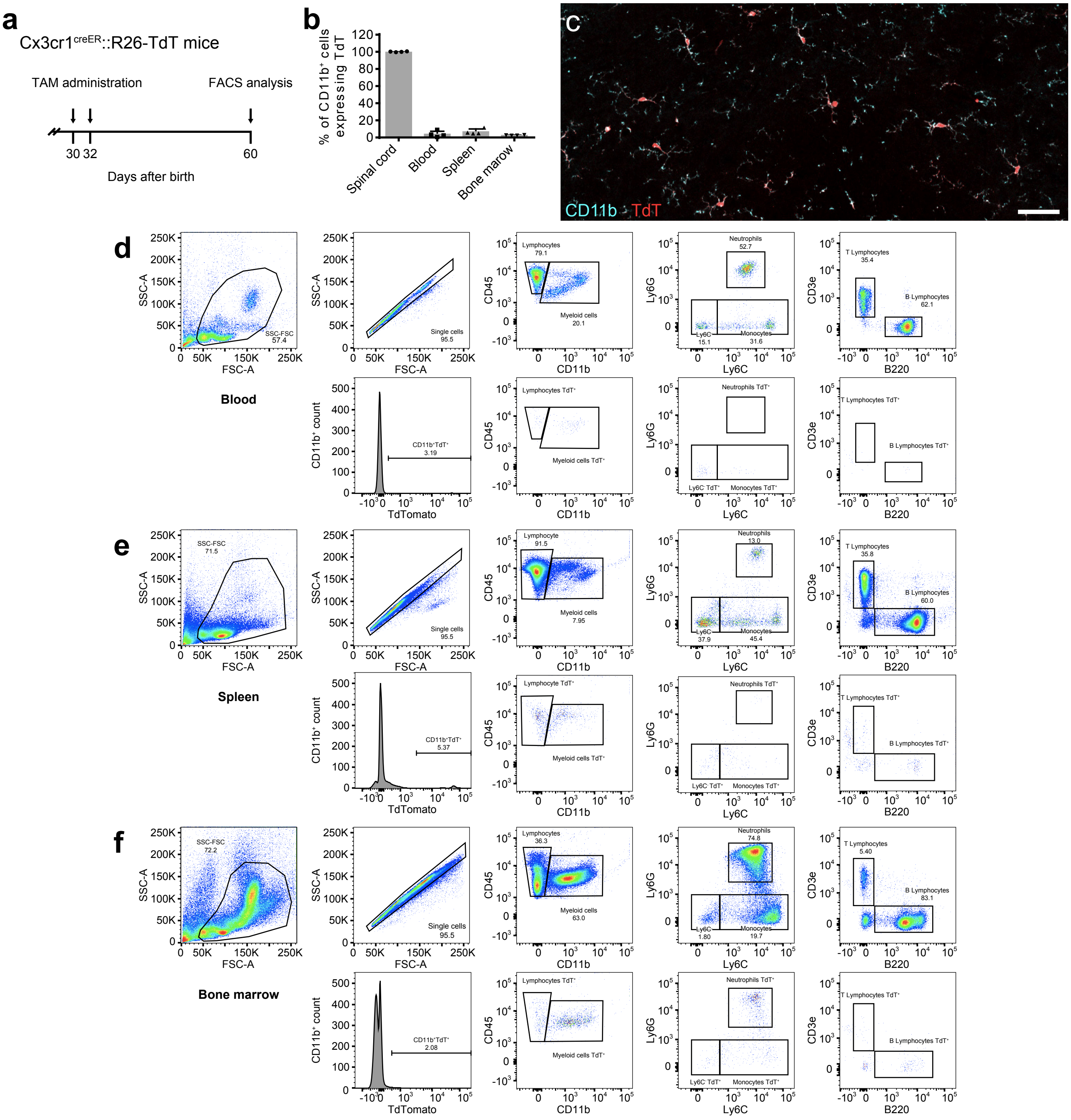

Supplementary Figure 1 | Adequate regimen of tamoxifen treatment in inducible *Cx3cr1*^{creER}::*R26*-TdT mice allows to specifically target microglia, while leaving monocytes and tissue-resident macrophages almost unaffected. (**a**) Schematic diagram of experimental design showing the timeline of tamoxifen (TAM) treatment and FACS analysis relative to the age of *Cx3cr1*^{creER}::*R26*-TdT mice. Transgenic mice were administered tamoxifen at postnatal day (P) 30 and 32 and then allowed to recover for 28 days prior to SCI to allow sufficient time for the turnover of MDMs and near disappearance of TdT^{+} cells in the blood, spleen and bone marrow. (**b**) Percentage of myeloid cells expressing the TdT fluorescent reporter 28 days following the last TAM injection. Note that virtually all CNS-resident myeloid cells are TdT^{+}, while only few (if any) myeloid cells in the blood, spleen and bone marrow express TdT (n=4 mice). (**c**) Confocal image showing the colocalization of CD11b (green) and TdT (red) proteins in the spinal cord of a *Cx3cr1*^{creER}::*R26*-TdT mouse at 28 days post-tamoxifen treatment. (**d-f**) Gating strategy used to identify immune cell subsets in the blood (**d**), spleen (**e**) and bone marrow (**f**) of *Cx3cr1*^{creER}::*R26*-TdT mice. For each panel from (**d**) to (**f**), the second row of plots shows the gating strategy used to identify TdT^{+} cells. **** p <0.0001, spinal cord versus peripheral fluids/tissues. Statistical analysis was performed using a one-way ANOVA followed by a Bonferroni's post hoc test. Scale bar: (**c**) 200 µm.

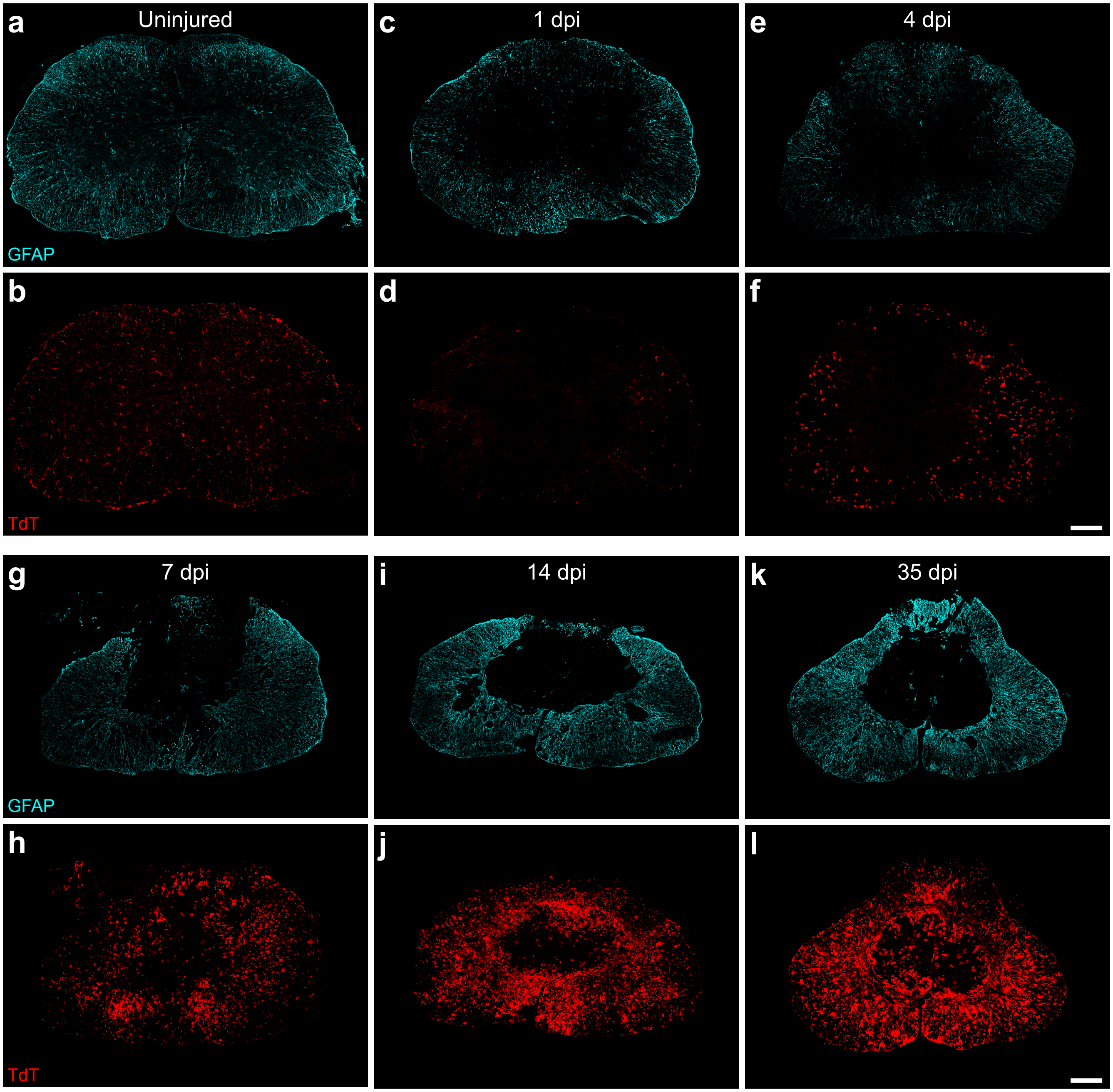

Supplementary Figure 2 | Microglia proliferate extensively and accumulate at the lesion border after SCI. (**a-l**) Individual color channels are displayed for the merged confocal images shown in Fig. 1a-f. Depicted are representative confocal immunofluorescence photomicrographs of spinal cord sections showing the spatio-temporal distribution of microglia (TdT, red) and astrocytes (GFAP, cyan) in an uninjured *Cx3cr1*^{creER}::*R26-TdT* transgenic mouse (**a-b**), as well as at the lesion epicenter at 1 (**c-d**), 4 (**e-f**), 7 (**g-h**), 14 (**i-j**), and 35 (**k-l**) days post-injury (dpi). Scale bars: (**a-l**, in **f** and **l**) 200 µm.

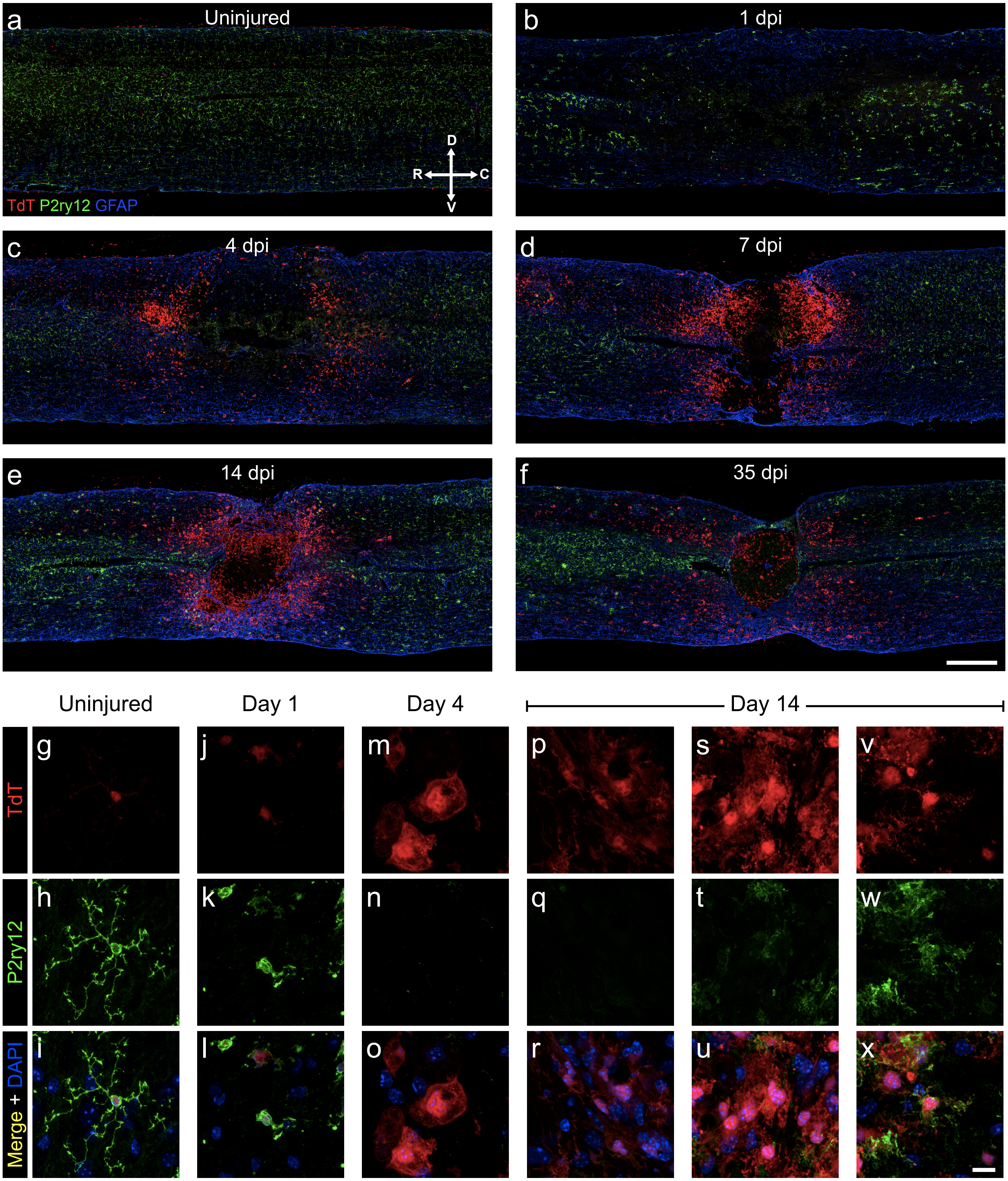

Supplementary Figure 3 | Microglia rapidly downregulate P2ry12 after SCI and regain expression over time. (**a**-**r**) Confocal immunofluorescence microscopy of representative sagittal sections showing the expression of P2ry12 (green) by TdT^+ microglia (red) in the uninjured spinal cord of *Cx3cr1*^{creER}::*R26*-TdT transgenic mice (**a, g-i**), as well as at the lesion epicenter in *Cx3cr1*^{creER}::*R26*-TdT mice killed at 1 (**b, j-l**), 4 (**c, m-o**), 7 (**d**), 14 (**e, p-x**), and 35 (**f**) days post-injury (dpi). Astrocytes ($GFAP^+$) are shown in blue in panels (**a-f**), while DAPI (blue) is shown instead in panels (**g-x**). Note the re-expression of the P2ry12 protein in some TdT^+ microglia at the lesion epicenter starting at day 14 post-SCI. Scale bars: (**a-f,** in **f**) 400 µm, (**g-x,** in **x**) 10 µm.

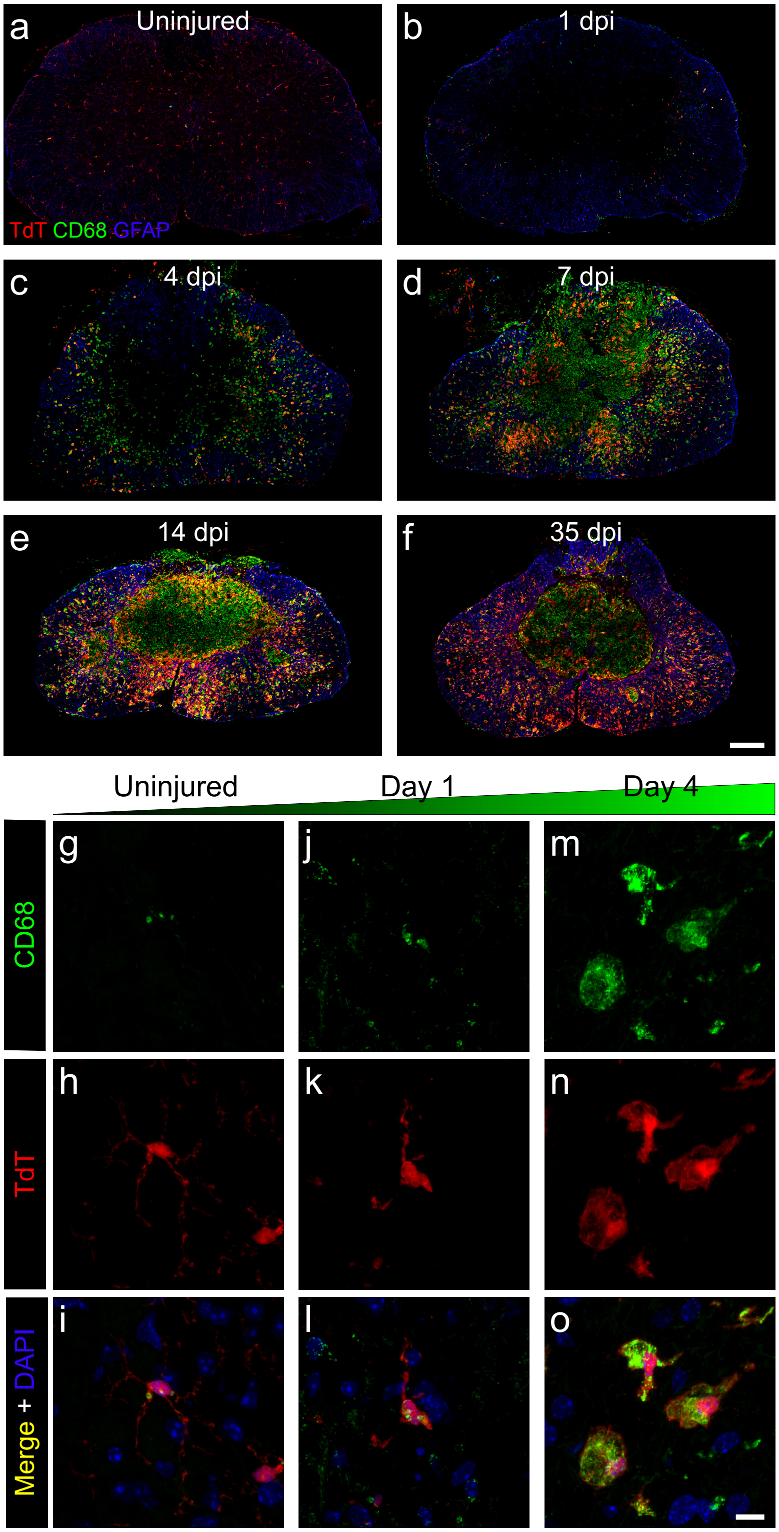

Supplementary Figure 4 | Microglia rapidly upregulate CD68 after SCI. (**a-o**) Confocal immunofluorescence microscopy of representative spinal cord sections showing the expression of CD68 (green) by TdT^{+} microglia (red) in the uninjured spinal cord of *Cx3cr1*^{creER}::*R26*-TdT transgenic mice (**a, g-i**), as well as at the lesion epicenter in *Cx3cr1*^{creER}::*R26*-TdT mice killed at 1 (**b, j-k**), 4 (**c, m-o**), 7 (**d**), 14 (**e**), and 35 (**f**) days post-injury (dpi). Astrocytes ($GFAP^{+}$) are shown in blue in panels (**a-f**), while DAPI (blue) is shown instead in panels (**g-o**). Scale bars: (**a-f,** in **f**) 200 µm, (**g-o**, in **o**) 10 µm.

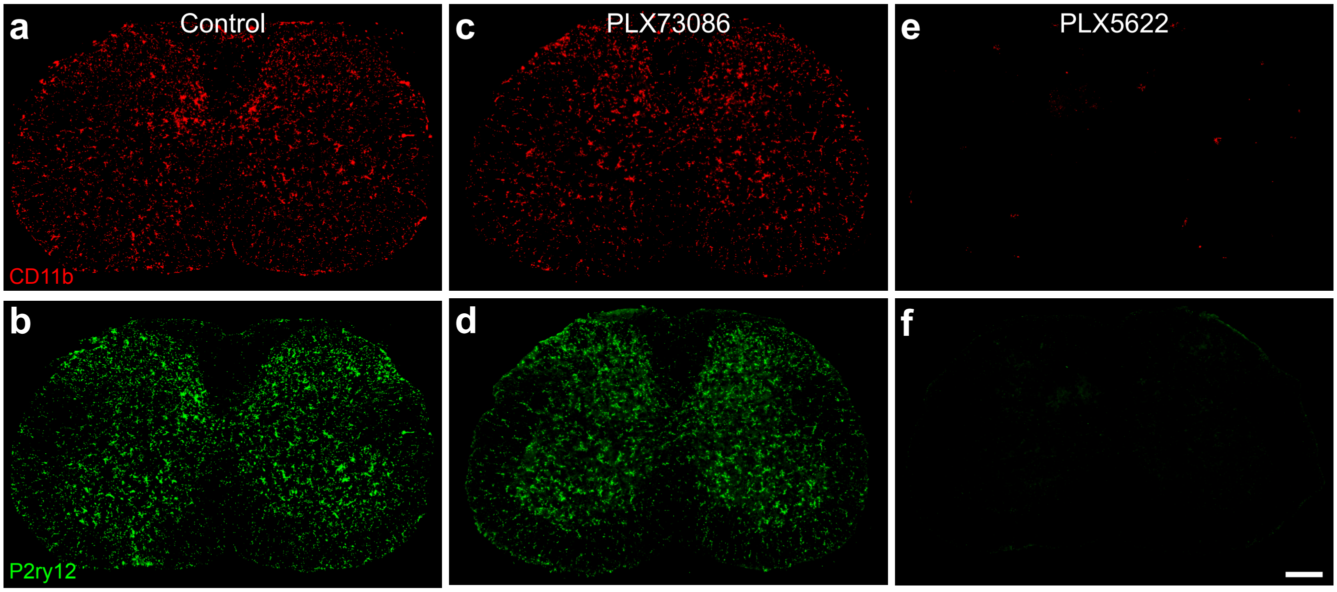

Supplementary Figure 5 | The CSF1R inhibitor PLX5622, but not PLX73086, crosses the intact blood-spinal cord barrier to deplete virtually all microglia. (**a-f**) Individual color channels are displayed for the merged confocal images shown in Fig. 2a-c. Depicted are representative confocal images of CD11b and P2ry12 immunostainings showing the almost complete elimination of microglia in the spinal cord of naïve (uninjured) C57BL/6 mice after treatment with the CSF1R inhibitor PLX5622 compared to those fed PLX73086 or the control diet. Mice were killed after 21 days of treatment. Scale bar: (**a-f**, in **f**) 200 µm.

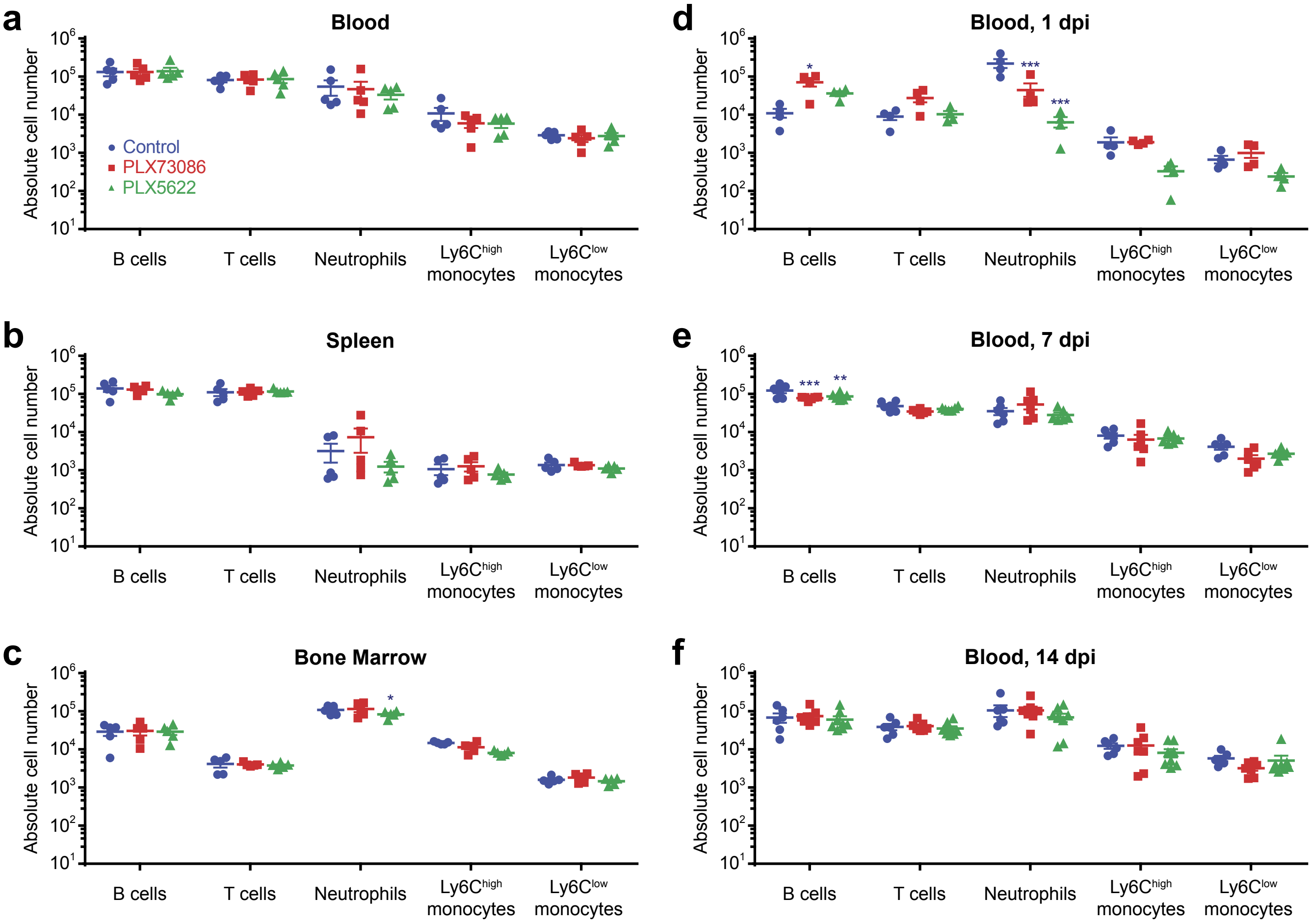

Supplementary Figure 6 | Treatment with CSF1R inhibitors barely affects the number of peripheral immune cells. (**a-f**) Absolute numbers of immune cells in the blood (**a, d-f**), spleen (**b**), and bone marrow (**c**) of uninjured (**a-c**) and spinal cord injured (**d-f**) mice following 3 weeks of treatment with either PLX5622, PLX73086 or control diet (n=4-8 mice per group per time point). No changes in cell numbers were detected, except a small and transient blood neutropenia at day 1 post-SCI in mice fed CSF1R inhibitors. Note that treatments were continued until the time of sacrifice. Data are expressed as mean ± SEM. * p <0.05, *** p <0.001, compared to the control group. Statistical analysis was performed using a two-way ANOVA followed by a Bonferroni's post hoc test.

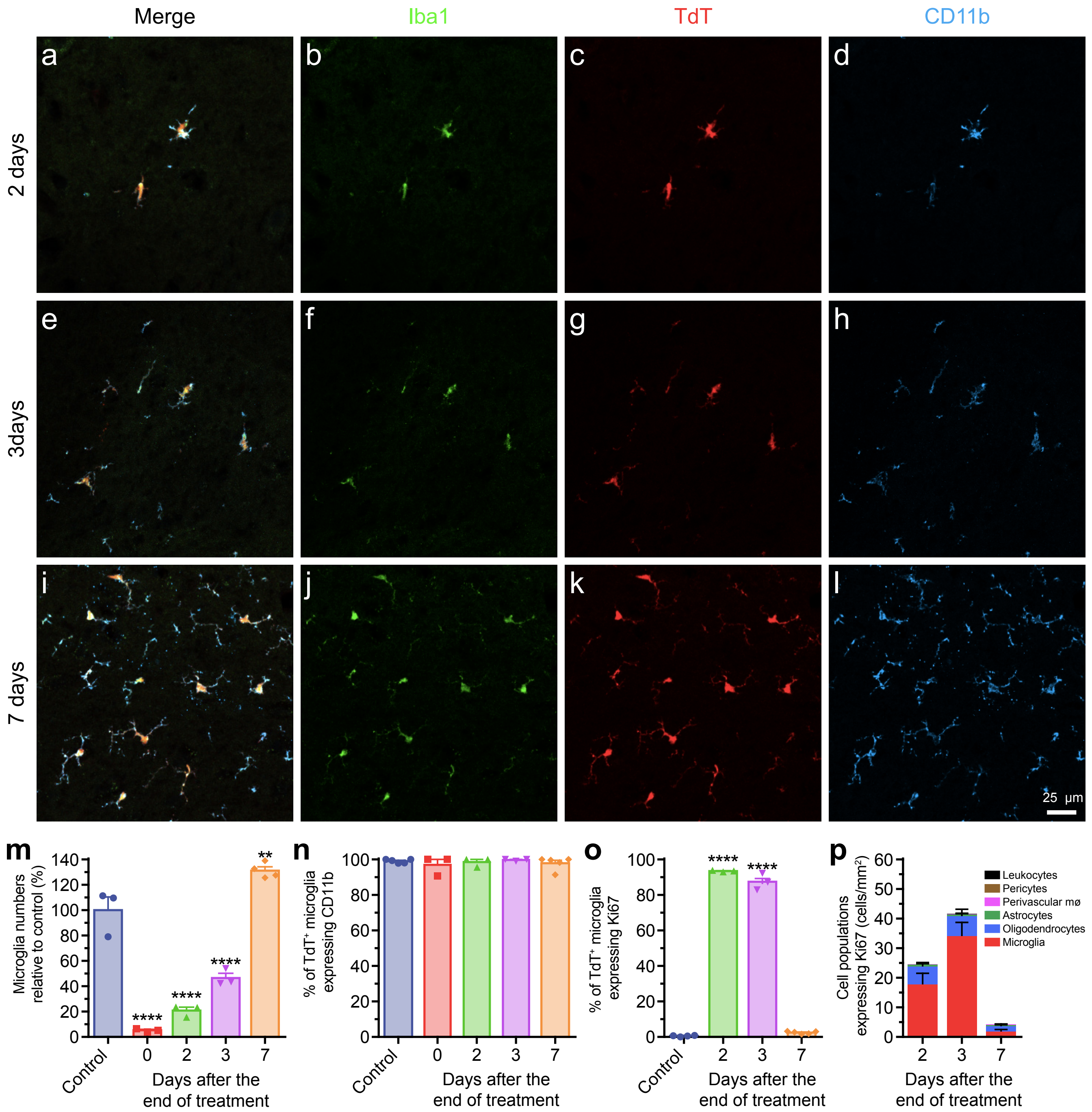

Supplementary Figure 7 | Microglia proliferate extensively and repopulate the entire spinal cord after one week of cessation of PLX5622 treatment. (**a-l**) Representative confocal images showing CD11b (blue), Iba1 (green), and TdT (red) signals in the uninjured spinal cord of *Cx3cr1*^{creER}::*R26*-TdT mice at 2 (**a-d**), 3 (**e-h**), and 7 (**i-l**) days after cessation of treatment with PLX5622. Nuclear staining (DAPI) is shown in turquoise. (**m**) Quantification of the number of TdT^{+} microglia in the normal thoracic spinal cord (control, blue bar), after 1 week of continuous treatment with PLX5622 (referred to as day 0 in the graph, red), as well as 2 (green), 3 (purple) and 7 (orange) days after cessation of PLX5622 treatment (n=3-5 mice per group and per time point). (**n-o**) Percentage of spinal cord microglia (TdT^{+}) expressing CD11b (**n**) and the proliferation marker Ki67 (**o**) at various times after cessation of PLX5622 treatment. (**p**) Number of microglia (TdT^{+}, red bars), oligodendrocytes (Olig2^{+}, blue), astrocytes (GFAP^{+}, green), perivascular macrophages (perivascular mø, CD206^{+}; purple), pericytes (CD13^{+}, brown), and blood-derived leukocytes (CD45^{+} TdT^{neg}, black) expressing Ki67 at various times after cessation of PLX5622 treatment. * $p < 0.05$, ** $p < 0.01$, *** $p < 0.001$, **** $p < 0.0001$, compared to the control group. Statistical analysis was performed using a one-way ANOVA followed by a Bonferroni's post hoc test. Scale bar: (**a-l**, in **l**) 200 μm.

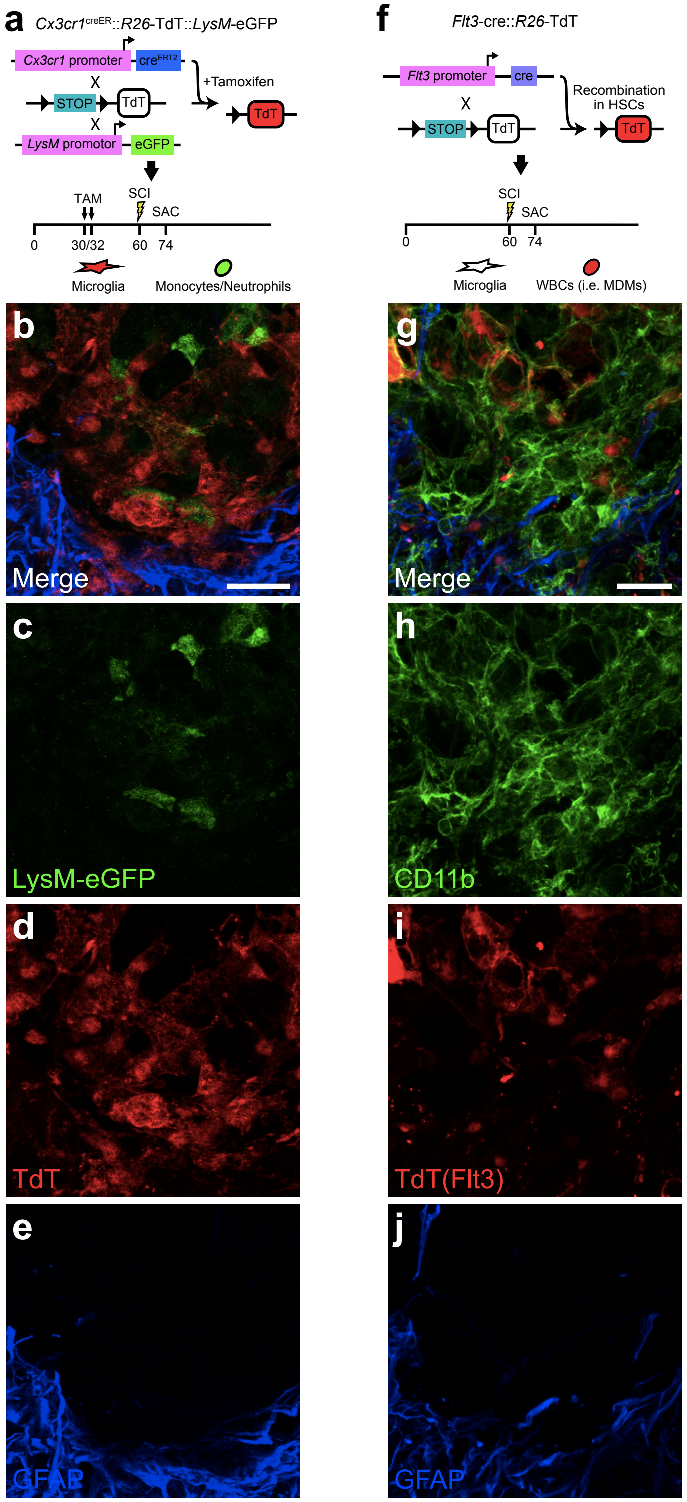

Supplementary Figure 8 | Fate-mapping analysis reveals that microglia form a scar between reactive astrocytes and infiltrated peripheral immune cells after SCI. (**a**) Schematic diagram showing the strategy of breeding to obtain *Cx3cr1^{creER}::R26-TdT::LysM*-eGFP mice, and the timeline of tamoxifen treatment, spinal cord contusion (SCI) and sacrifice (SAC). (**b-e**) Representative confocal images showing the microglial scar formed of TdT^{+} microglia (red cells), some of which are in direct contact with GFAP-immunoreactive astrocyte endfeet (blue) on one side and LysM-eGFP^{+} blood-derived myeloid cells (green cells) on the other side at 14 days post-SCI. (**f**) Schematic diagram showing the breeding strategy to obtain *Flt3-cre::R26-TdT* mice, in which TdT is expressed in hematopoietic stem cells (HSCs) and their progeny (e.g. monocyte-derived macrophages, MDMs), but not microglia. (**g-j**) Representative confocal images showing the microglial scar formed of CD11b^{+} Flt3^{neg} microglia (green cells), making close contact with astrocyte endfeet (GFAP^{+}, blue) and infiltrating MDMs (CD11b^{+} Flt3^{+}, green cells with red nuclei) at 14 days post-SCI. Scale bars: (**b-e**, in **e**) 20 µm, (**g-j**, in **j**) 20 µm.

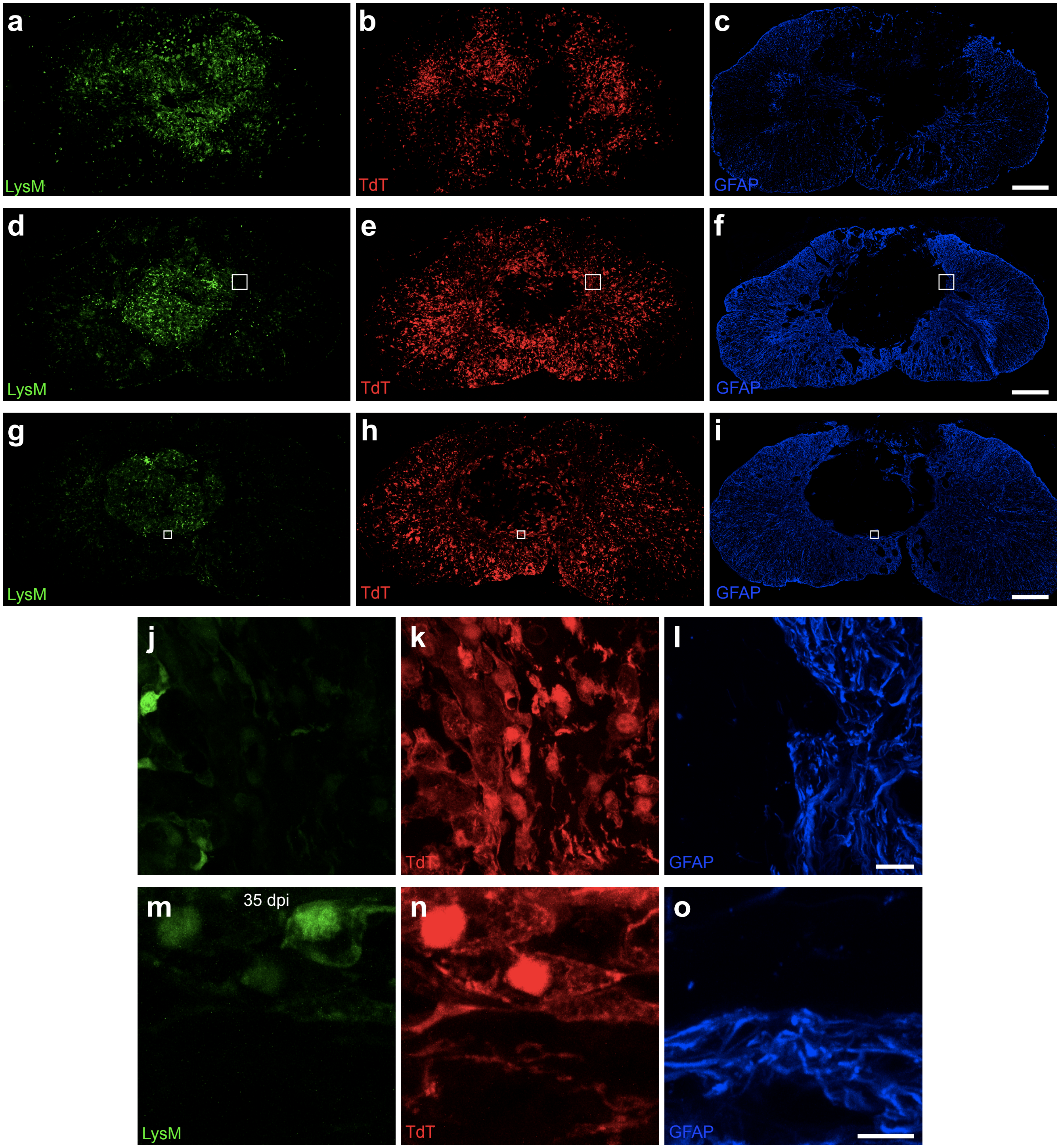

Supplementary Figure 9 | A microglial scar forms at the interface between reactive astrocytes and blood-derived myeloid cells that infiltrate the lesion site. (**a-o**) Individual color channels are displayed for the merged confocal images shown in Fig. 4a-e. Depicted are representative confocal immunofluorescence photomicrographs of spinal cord sections taken at the lesion epicenter at 7 (**a-c**), 14 (**d-f, j-l**), and 35 (**g-i, m-o**) days post-injury (dpi) showing formation of the microglial scar, characterized by the accumulation of TdT^{+} microglia (red) at the lesion borders, over time. The microglial scar is shown in relation to the infiltration of blood-derived myeloid cells (LysM-$eGFP^{+}$, green) and formation of the astroglial scar (GFAP-immunoreactive astrocytes, blue). Panels (**j-l**) and (**m-o**) are insets of panels (**d-f**) and (**g-i**), respectively, showing close-ups of the microglial scar in *Cx3cr1*^{creER}::*R26-TdT*::*LysM*-eGFP mice at 14 and 35 dpi. Scale bars: (**a-i**) 200 μm; (**j-o**) 20 μm.

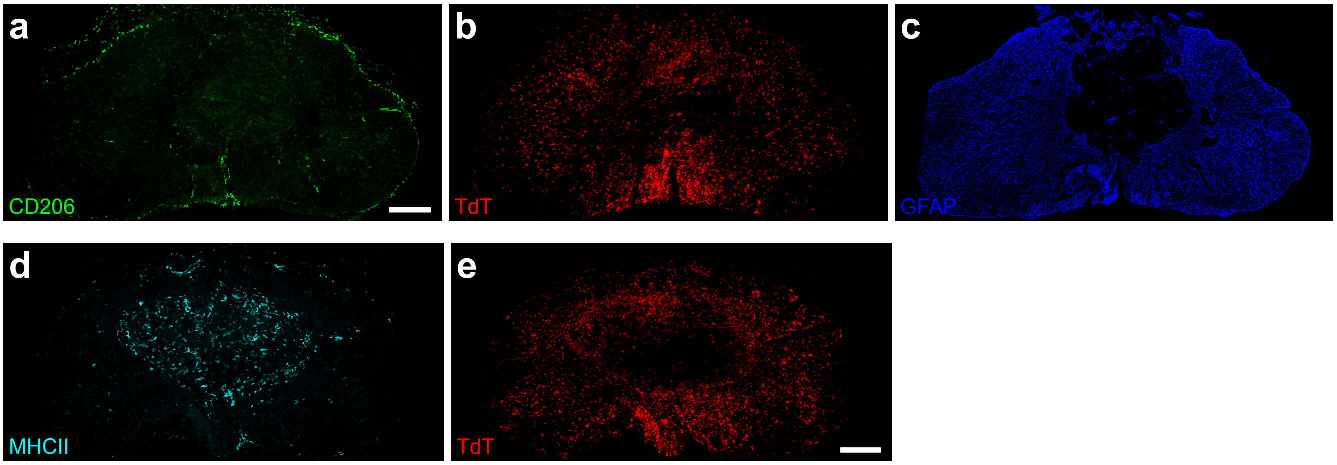

Supplementary Figure 10 | The microglial scar is mainly composed of microglia with the presence of only few scattered CNS border-associated macrophages. (**a-c**) Individual color channels are displayed for the merged confocal image shown in Fig. 5i. Depicted are representative confocal immunofluorescence photomicrographs showing the absence (or very weak expression) of CD206 (green) in microglia (TdT^{+}, red) forming the microglial scar at the lesion borders at 14 days post-SCI. In contrast, border-associated macrophages express high levels of the CD206 protein. (**d-e**) Individual color channels are displayed for the merged confocal image shown in Fig. 5p. Depicted are representative confocal image showing the absence of colocalization between TdT (red) and MHCII (cyan) in the injured spinal cord of a *Cx3cr1*^{creER}::*R26-TdT* mouse at 14 days. Scale bars: (**a-c**, in **c**) 200 µm, (**d-e**, in **e**) 200 µm.

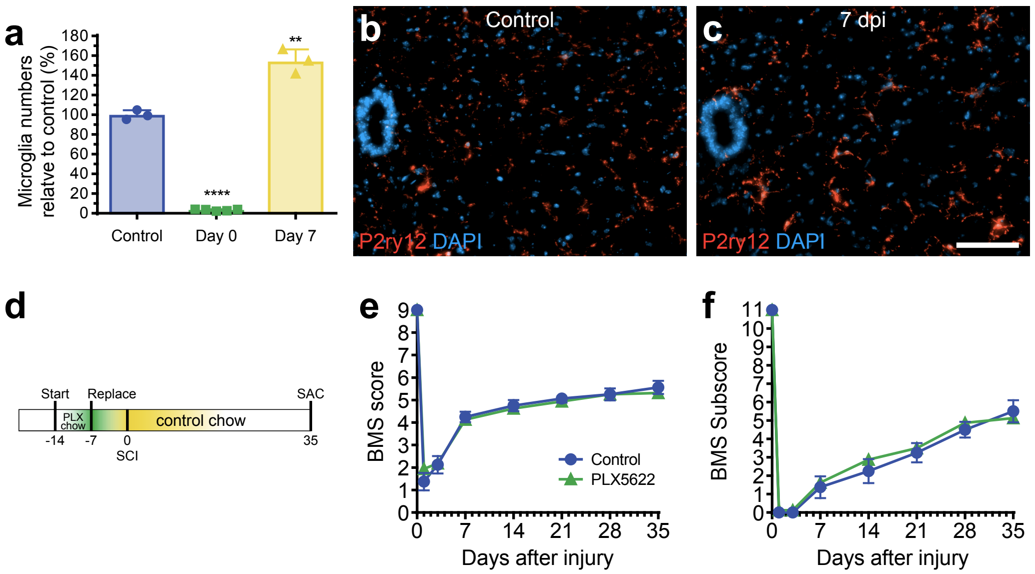

Supplementary Figure 11 | Mice with more microglia in their spinal cord at the time of injury recover locomotor function similar to that of SCI mice on the control diet. (**a**) Quantification of the number of microglia (P2ry12^{+}) in the uninjured thoracic spinal cord of C57BL/6 mice (control, blue bar in the histogram), as well as in mice treated with PLX5622 for 1 week and then switched to the control diet for 0 (green) or 7 (yellow) days (n=3-5 mice per group). (**b-c**) Representative confocal images of P2ry12 immunostaining showing the increased number of spinal cord microglia after switching C57BL/6 mice from the PLX5622 diet to control chow for 7 days. (**d**) Schematic diagram of experimental design showing the timeline of treatment (PLX5622 versus control chow), spinal cord contusion (SCI) and sacrifice (SAC). (**e-f**) Assessment of locomotor recovery using the BMS (**e**) and BMS subscore (**f**) (n=8 mice per group). ** $p < 0.01$, **** $p < 0.0001$, compared to the control group. Statistical analysis was performed using a one-way ANOVA (**a**) or a two-way repeated-measures ANOVA (**e-f**) followed by a Bonferroni's post hoc test. Scale bar: (**b-c,** in **c**) 50 µm.

Supplementary Table 1. List of cDNAs used for *in situ* hybridization.

					Linearization		Transcription	
**Symbol**	**Gene Name**	**Genbank Accession Numbers**	**Position**	**Size (bp)**	**Antisense**	**Sense**	**Antisense**	**Sense**
Igf1	Insulin-like growth factor 1	NM_184052	1338-1856	868	HindIII	XhoI	T7	SP6
Il1a	Interleukin 1 alpha	NM_010554	270-1985	1715	XhoI	BamHI	SP6	T7
Il1b	Interleukin 1 beta	M15131	1-1339	1358	KpnI	XhoI	T7	SP6
Il6	Interleukin 6	J03783		600	KpnI	HindIII	T3	T7
Tnf	Tumor necrosis factor	NM_013693	426-844	419	XhoI	HindIII	SP6	T7
Tgfb1	Transforming growth factor, beta 1	BC013738	315-1487	1173	EcoRI	XhoI	T3	T7
