# Supplementary material for: Microglia are an essential component of the neuroprotective scar that forms after spinal cord injury
Source: Nat Commun. 2019 Jan 31;10:518. doi: 10.1038/s41467-019-08446-0 (PMC6355913; doi:10.1038/s41467-019-08446-0)
Supplement: Supplementary file 2 — Description of Additional Supplementary Files [file 41467_2019_8446_MOESM2_ESM.docx]

**Description of Additional Supplementary Files**

File Name: Supplementary Movie 1

Description: Morphology of a dividing microglia at the lesion epicenter after spinal cord injury. Animated 3D reconstruction of a confocal XYZ stack of images showing a dividing microglia (TdT+, red) immunostained for the proliferation marker Ki67 (green) in the spinal cord of aCx3cr1creER::R26-TdT mouse at 4 days after spinal cord injury (SCI). The nuclear staining 4',6-diamidino-2-phénylindole (DAPI, blue) shows the segregation of the DNA material at the two poles ofthe TdT+ microglia, indicating that the cell is in the telophase stage of cell division.
